# The “discordant doppelganger dilemma”: SGLT2i mimics therapeutic carbohydrate restriction - food choice first over pharma?

**DOI:** 10.1038/s41371-021-00482-y

**Published:** 2021-02-09

**Authors:** Scott W. Murray, Sean McKelvey, Thomas D. Heseltine, George Henderson, Jagdeep Singh, David Unwin, Adrian J. B. Brady

**Affiliations:** 1 Wirral University Teaching Hospital, Wirral, UK; 2 Liverpool Centre for Cardiovascular Science, Liverpool, UK; 3 Institute for personalized therapeutic nutrition, Kelowna, BC Canada; 4 PreKure, Auckland, New Zealand; 5 Edinburgh Royal Infirmary, Edinburgh, UK; 6 The Norwood Surgery, Southport, UK; 7 University of Glasgow, Glasgow, Scotland UK

**Keywords:** Renovascular hypertension, Fat metabolism, Heart failure

Type 2 Diabetes is a global emergency threatening to take more lives (as a root cause of ill-health) than world wars, global infectious pandemics and terrorism combined [1, 2]. The pharmaceutical industry has therefore recognised this as being a strong market and subsequently many lucrative therapies have been developed recently in the cardio-metabolic paradigm, such as the Sodium Glucose Co-Transport-2 Inhibitors (SGLT2i). At the European Society of Cardiology Annual Conference in 2019, the results of the SGLT2i Dapagliflozin trial DAPA-HF were released with great expectations [3] The following year, the full results of EMPEROR-Reduced were presented at the ESC Congress 2020 and showed again that a relative risk reduction of 25% was possible in those with and without diabetes, this time with Empagliflozin [4]

This class of drugs appears to have profound benefits across both diabetic patients and “non-diabetic” patients, in relation to the soft cardiovascular end-points. The most benefit appears to be in heart failure, with a mechanism purported to be related to a diuretic effect and blood pressure lowering as a result, although many other mechanisms are being currently explored [5]. These drugs have already achieved the highest ranking in the latest ESC guidelines (1A). This same guideline barely mentions dietary change, other than low calorie and low-fat options [6]. See Fig. 1.

**Fig. 1:**
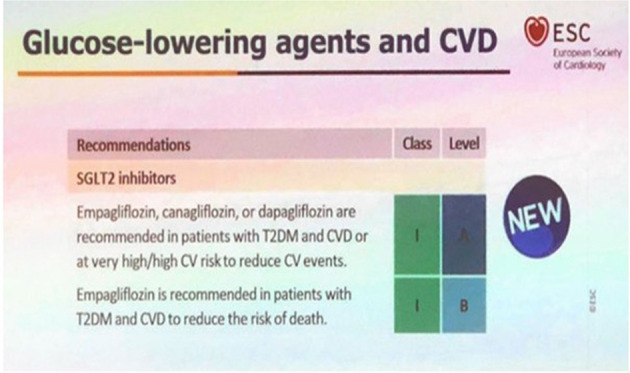
The latest ESC guidelines for glucose lowering agents and CVD - SGLT2i hold a coveted 1A rating.

Concurrently, some of the above authors have recently published that adopting a carbohydrate restricted diet can have profound benefits in the control of diabetes; blood pressure; weight and some lipid markers [7]. In order to explain this BP reduction phenomenon, a potential deeper mechanism at the level of the kidney vasculature became clearer during some research into the effects of the SGLT2i drugs. The most convincing theory is that seen in randomised controlled trials [8]. These drugs appear to improve multiple endpoint outcomes across diabetes, cardiovascular disease and renal medicine [8, 9]. Looking at this in more depth, hyperglycaemia causes hyperfiltration and the intra-glomerular pressure to rise [10, 11]. Virtually all glucose filtered at the glomerulus is reabsorbed in the proximal convoluted tubule (PCT), up to a limit of around 180 mg/dL or 220 mg/dl in diabetic patients [10]. Above this level the maximal re-absorptive capacity is breached and excess glucose appears in the urine. SGLT2 is responsible for 80–90% of this reabsorption, with SGLT1 (a related transport system) in the distal part of the PCT responsible for the remainder (10–20%) [10]. Hyperinsulinaemia augments the expression of SGLT2 in a dose-dependent manner and therefore the capacity within this system, which promotes a vicious cycle of increased ability to ‘save’ not just glucose but also sodium [11, 12]. This therefore maintains and exacerbates hyperglycaemia and sodium/fluid retention, worsening diabetic control and hypertension. Moreover, hyperglycaemia disrupts delicate tubule-glomerular feedback mechanisms, reducing sodium delivery to the macula densa, mimicking kidney hypo-perfusion [10, 11]. This leads to afferent arteriolar dilatation, efferent arteriolar constriction and worsening intra-glomerular hypertension, thereby accelerating nephropathy [10, 11]. SGLT2 inhibitor drugs (Empagliflozin, Dapagliflozin and Canagliflozin) reverse the above mechanisms, preventing sodium and glucose reabsorption and allowing normal tubulo-glomerular feedback to occur [8–11]. It can therefore be hypothesised that reducing hyperinsulinaemia and post-prandial hyperglycaemia by certain dietary strategies, naturally reduces SGLT2 expression and up-regulation in the kidney, without the need for drugs. SGLT2i drugs are known to cause an osmotic diuresis and a contraction in plasma volume, estimated to be around 7% [7–10]. This diuretic-like effect is mentioned by patients, as it is seen in carbohydrate restricted diets with a certain amount of glycogen related “water-weight” being lost in the initial stages” [13].

In addition to the above described mechanisms, on a daily basis over 500 g of sodium is filtered via the kidneys. Collectively, the proximal tubules are responsible for reabsorbing up to 70% of this filtered sodium [14]. In a normal physiological state, it is estimated that only 6% of sodium is reabsorbed via SGLT2 and little, if any, via SGLT1 [15]. However, when blood glucose levels increase, as is seen post-prandially following ingestion of sugar/starch and permanently in diabetic states, it is estimated that up to 22.8% of the sodium reabsorbed in the proximal tubules is retained as a direct result of SGLT2 activity [15]. The total amount of sodium reabsorbed can be calculated since the molar ratio of glucose to sodium is 1:1 for SGLT2. The maximal SGLT2 glucose transport in patients with type 2 diabetes has been estimated at 500–600 g/d, representing a total of 2.75–3.3 moles of glucose (180 g/mol) [16] This reflects a maximal SGLT2 sodium reabsorption total of ~63–76 g/d (1 mole of sodium weighs 22.99 g). The Brady et al model [15] predicts approximately 80 g/d (22.8% × 70% × 500 g/d).

The role of SGLT1 in sodium retention is not currently known but can be estimated since approximately 10–20% of glucose (up to 100 g max/day) is reabsorbed via this transporter [16]. SGLT1 follow a 2:1 molar ratio of sodium to glucose which effectively doubles the sodium being reabsorbed when the SGLT2 threshold is exceeded [17]. Assuming saturation of SGLT1 in uncontrolled diabetes we can convert 100 g of glucose to moles (100 g/180 g mol^−1^ = 0.55 mol). Doubling this would result in 1.1 moles or ~25 g (22.99 g/mol × 1.1 mol) of reabsorbed sodium. Therefore, in the context of hyperglycaemia, the combined effect of sodium glucose transporters could be responsible for up to 100 g of the sodium reabsorbed in the proximal tubules.

Recent research into the role of SGLT2 inhibitors has also established a direct connection between SGLT2 transporters and NHE3 activity (an important Na and bicarbonate reabsorption process) [18]. Inhibiting SGLT2 significantly reduces activity of NHE3 which results in increased HCO3 loss in the urine. This loss of buffering capacity combined with reduced insulin (and increased lipolysis) is one of the key reasons why euglycemic diabetic keto-acidosis (DKA) is possible with SGLT2 inhibitors in the context of an acute illness. For the purpose of sodium retention, it also shows that SGLT2s directly influence NHE3 resulting in additional sodium retention through a separate but related mechanism.

Now that we know there is both a direct and indirect role for SGLT 2 and 1 on sodium retention and that glucose load is one of the key determinants, we hypothesise that a large percentage of “essential” hypertension could be treated with avoiding post-prandial and permanent hyperglycaemia, using therapeutic carbohydrate restriction, as seen in our previous paper [7].

## Salt and fat vs sugar and starch – lipid energy or lipid locked away?

**Fig. 2:**
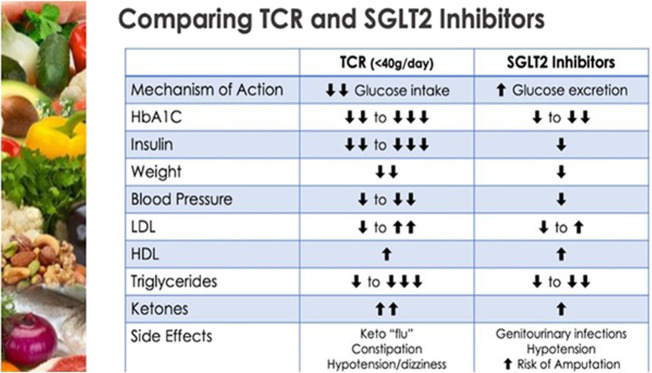
Table comparing Therapeutic Carbohydrate Restriction and SGLT2 inhibiton with similar effects on traditional CVD risk factors.

So, have we been blaming excess salt ingestion, for what the sugar and simple starch also does? Or should we simply say avoid frequent post-prandial (or any) hyperglycaemic insults and more significantly the perpetual hyperglycaemia of diabetes? SGLT2i are similar to some dietary changes in that they are inherently ketogenic due to their mode of action [19, 20] they decrease glucose oxidation in favour of fat oxidation and promote free fatty acid utilisation. Losing glucose from the body in the urine and its subsequent stunted abundance, promotes glucagon over insulin and the mobilisation of glycogen stores with gluconeogenesis initially, followed by the imminent need to switch to fat oxidation for fuel. Moreover, for the first time, it is being recognised that these drugs raise the level of measurable Low Density Lipoprotein (LDL) in the circulation. Despite this being historically portrayed as “bad cholesterol” these drugs still have profound benefits in terms of cardiovascular outcomes [21] It is Important to note that these drugs increase both LDL and HDL so the lipoprotein ratios, that are more powerful in risk prediction than LDL alone, remain unchanged. The increase in LDL-C levels related to SGLT2 inhibitors is trivial, while at the same time they may induce a mild decrease in LDL particle number, switching their profile from type B towards type A. This is due to higher Lipoprotein Lipase (LPL) activity, possibly due to decreased ANGPTL4 expression. Moreover, these drugs mimic therapeutic carbohydrate restriction by reducing the triglyceride level and improving the triglyceride to HDL ratio, this is all despite the potential for overall maintenance of calorific intake but significantly reduced glucotoxicity [22] See Fig. 2.

It is also being recognised that transient fasting, can raise the LDL level in certain individuals, prolonged fasting and the resulting cachexia are associated with hypolipidemia and low LDL-C. However, weight loss in metabolically obese individuals, with high VLDL levels can be associated with an LDL rise, due to more effective conversion mediated by LPL. Intriguingly, some individuals with lipodystrophy, low personal fat thresholds and anorexia nervosa have very high LDL-C levels. If LDL changes occur during overall metabolic improvements and weight loss, similarly to SGLT2i, by shifting the metabolism towards lipid/ketone utilisation and away from lipogenesis [23–25] How will this affect the lipid hypothesis of cardiovascular disease? The simplistic approach of using LDL-C as the proxy of the total load of proatherogenic ApoB particles, including remnants of TGRLs, significantly contributes to the risk in patients with the metabolic syndrome and familial lipid disorders. However, could this also be a sign that the lipid system is intricately involved in the transition of energy around in the blood and it is therefore unlikely that this evolutionary mechanism, that creates native LDL cholesterol, is inherently toxic to cardiovascular health? This could be explained by the oxidised, glycated, small dense LDL particle being the key driver within proatherogenic total ApoB [26–28]

The medical media driven fear of “cholesterol” in the blood and therefore, fat in the diet, has driven the low-fat dietary paradigm. This is despite the fact that there is not a linear relationship between the consumption of “fat”, or saturated fat, and the level of “fat”, as triglycerides, or as saturated fat, in the bloodstream [27, 28]. Certainly, the consumption of excess calorific energy in the form of carbohydrates and specific fats together could contribute to the elevation of “blood fats” such as palmitic acid, via the combined effect of hepatic de novo lipogenesis and subsequent reduced rates of lipid oxidation or storage creating spill back of non-esterified fatty acids into the blood. Moreover, the knowledge that cholesterol synthesis via HMG-CoA reductase is kept over-activated by hyper-insulinaemia and stopped by glucagon, ensures that the liver cholesterol synthesis rate will be reduced in low insulin states [28]. The same is true for macrophage cholesterol retention, which is also somewhat insulin-dependent [29].

### SGLT2i benefits in heart failure

The harmful glucotoxicity and lipotoxicity experienced in end stage type 2 diabetes creates an energetic crisis at the mitochondrial level with excess glucose being stored as fat in an environment already swamped with abundant FFA and a paradigm that has promoted glucose utilisation over fat. With regards to myocardial energetics and the benefits we see from these drugs in heart failure, is there a mechanism over and above losing salt/glucose in the urine creating a diuretic effect and the down regulation of renin release lowering blood pressure? Can the switch to improved FFA and ketone utilisation potentially help the heart “burn” its way out past this “sea” of excess substrates? In the healthy heart, cardiomyocytes are able to freely switch between FFA, glucose, lactate, ketones and other fuel substrates depending on availability, workload and tissue perfusion [30]. In a state of rest, the healthy heart predominantly uses long chain FFA as a fuel substrate because of its dense energy content. However, during periods of increased workload, glucose can be utilised as it is a more oxygen-efficient fuel; although it may liberate less energy per molecule compared to FFA, it has a better energy yield per oxygen atom consumed. Similarly, under hypoxic conditions such as myocardial ischaemia, LVH, or in the failing myocardium, there is an adaptive response that favours glucose as the primary fuel substrate via enhanced glucose uptake, activation of glycolytic pathway enzymes and ultimately a reduction of FFA oxidation [30, 31]. By switching the fuel substrate, the heart is able to reduce the supply-demand mismatch and temporarily improve myocardial contractile efficiency by increasing the energy production-to-oxygen consumption ratio. However, by primarily burning glucose in a situation of excess, lipid may further accumulate and with FFA oxidation downregulated, the situation of lipotoxicity may worsen [32].

Concurrently, in patients with insulin resistance and T2DM, the ability to switch to a predominantly glucose fuel substrate can also be lost. Insulin resistance reduces the efficiency of the GLUT-4 glucose transporter protein which is responsible for glucose uptake into the cell [33]. Additionally, there continues to be increased delivery of FFA as a result of enhanced lipolysis and de novo lipogenesis with liver, muscle and adipocyte insulin resistance [34]. Longstanding elevation in intra-myocyte FFA levels activate the nuclear receptor peroxisome proliferator-activated receptor-alpha (PPAR- α), which in turn results in further mitochondrial FFA transport and attempted oxidation; thereby trying to fix the myocyte into a FFA-based fuel substrate use. Excess FFAs further impairs the intracellular insulin signalling pathways, consolidating the insulin resistance of the myocyte and relegating potentially more efficient glycolytic pathways even further.

Apart from predisposing to an adverse myocardial energetic profile, glucotoxicity and lipotoxicity is another potential factor contributing to diabetic cardiomyopathy. When myocardial FFA uptake outpaces its β-oxidation capacity, the excess FFA is converted to triacylglycerol (TAG) leading to cardiac steatosis. Additionally, there is also accumulation of potentially toxic by-products of β-oxidation such as diacylglycerol (DAG) and ceramide [35]. This intra-myocyte build-up of TG, DAG and ceramide generates Reactive Oxygen Species (ROS). Early changes of ROS damage are seen in the sarcoplasmic (endoplasmic) reticulum, where there is inactivation of its calcium-ATPase [36]. This results in reduced sequestration of calcium in the sarcoplasmic reticulum and a cytosolic calcium overload, which in turn causes myocardial fibrosis, hypertrophy and diastolic dysfunction [37]. Further accumulation of ROS induces mitochondrial uncoupling (dysfunction) and eventually apoptosis [30]. The very substrates the heart was meant to utilise in health become a burden in a state of energy toxicity and therefore by potentially enhancing whole body glucose deprivation (reduce glucotoxicity) and increasing FFA utilisation (reduce lipotoxicity) using either SGLT2i or therapeutic carbohydrate restriction, there appears to be a theoretical solution to this increasingly common myocardial energy crisis, could the ketone body Beta Hydroxybutyrate (BHb) be the missing link here? More research is required.

**Fig. 3:**
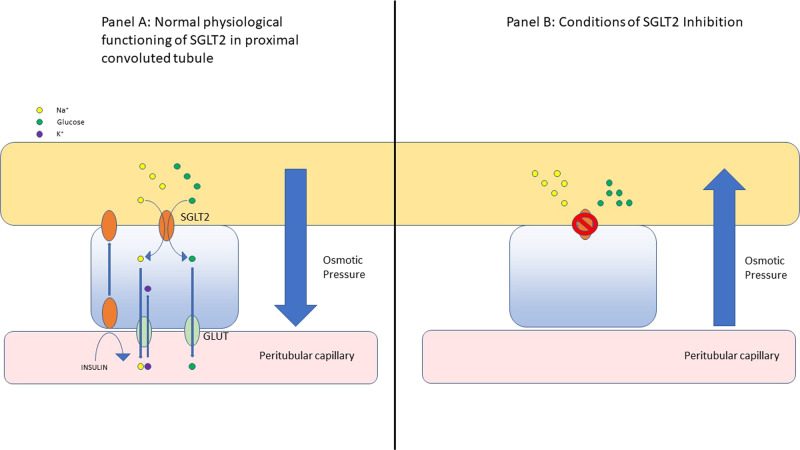
Infographic of the interplay between solutes, the renal tubule and blood capillary, from the perspective of SGLT in normal physiology and SGLT2 inhibition.

**Fig. 4:**
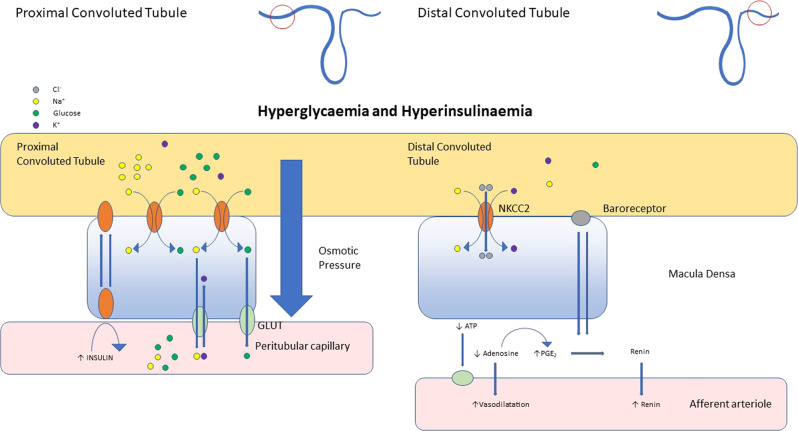
The effects of hyperglycaemia and hyperinsulinaemia on SGLT2 performance in the kidney tubule and arterioles.

**Fig. 5:**
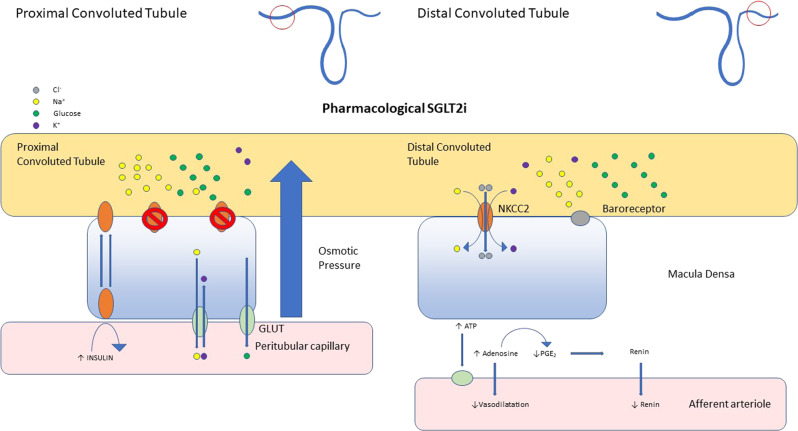
The effects of pharmacological SGLT2 inhibition in a diabetic model of the kidney tubule and arteriole.

**Fig. 6:**
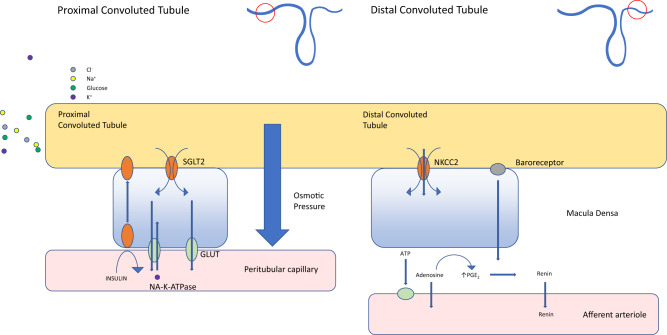
JPEG file schematic of the movement and transfer of solutes from the kidney tubules to capillaries. The role of SGLT2.

Interestingly, the drug Ranolazine is a myocardial fatty acid oxidase inhibitor and it has shown benefits in terms of angina control, reduction of Hba1c in diabetics and therefore this may also be able to help address the substrate problem of glucotoxicity or to specifically decrease fatty acid oxidation in heart failure. While also considered to be an inhibitor of the late Na+ current, ranolazine is capable of activating PDH a rate limiting enzyme for glucose oxidation [37]. However, ranolazines efficacy in treating heart failure has not been extensively studied. Whatever the final mechanism is at the cellular level, the latest clinical endpoint results for SGLT2i that increases salt and glucose loss, promoting fat oxidation and beta hydroxybutyrate levels, appears to be most profound. More study here is warranted due to the physiological complexity, as evidence by this recent review [38] See Figs. 3–6 for the effects on the kidney.

### Therapeutic carbohydrate restriction (TCR) – the Norwood diet

With regard to utilising dietary changes and lifestyle, if we know SGLT2i waste glucose from the body, then why not simply avoid consuming excess glucose in the form of free sugar or simple starch? Several studies and programs in this arena of therapeutic carbohydrate restriction or ketogenic nutrition have shown that this can promote very tight glycaemic control, enhanced time in range and the “remission” of type 2 diabetes [7, 39, 40].

So far Dr David Unwin in the UK has achieved a 50% drug-free remission rate of type 2 diabetes utilising TCR in selected individuals [7].

Intermittent fasting or calorie restriction in the form of very low energy diets (VLED) can also do the same as above. The DIRECT trial and previous work by Taylor et al. have shown us that the metabolic effects of utilising VLED to facilitate the loss of subcutaneous and visceral fat can create room within adipocytes and this removal of body fat with reduction in weight and BMI are highlighted as the main goals to achieve diabetes remission [41]. Clearly the metabolic effects may go beyond simple weight loss and this oversimplification may lead patients to consider the continued use of processed food or meal replacement shakes at lower calorific densities, as the only way to lose weight. While lifestyle changes should be the base of primary and secondary ASCVD prevention, all available evidence points out that (sadly) weight loss associated with lifestyle changes and diet is temporary for many and over time more patients regain most of the lost weight. Only bariatric surgery or long-term use of medications have shown enduring effects. Conversely, SGLT2i may actually stimulate caloric intake to maintain energy balance, whilst still achieving weight loss and soft cardiovascular end point results. Clearly more research is needed here for both TCR and SGLT2i.

## Conclusions and recommendations

Where the Discordant Doppelganger Dilemma occurs in clinical practice is when the SGLT2i drugs are always chosen as first line, as per the latest ESC guidance. We then may potentially lose the chance to leverage diet and lifestyle changes first (TCR and VLED). The possible risk of hypoglycaemia or euglycaemic ketoacidosis exists when combining significant dietary change with SGLT2i. The physiology has been described as far as we know it earlier here and is constant and reproducible. The drugs work in highly controlled clinical trials but can have side effects and the lifestyle changes work in observational trials and specialist centres, but could be hard to maintain for everyone. So how can we get the drug-like outcomes, without financing or taking the drug? What is the right way to move forward when both can produce “mirror-like” results but they cannot therefore be combined? In a way we are lucky to have new options to improve the outlook for these patients but there remains a pertinent analogy: If we found out tomorrow that cancer could be prevented in 50% of people (or put into drug-free “remission”) by avoiding the ingestion of known substances, but at the same time an expensive drug could eliminate those substances from the body, what would be the best course of action for everybody? Taking this from a philosophical, moral, ethical, economic and human perspective? Should one avoid the known substances or keep ingesting the substances whilst concurrently eliminating them with the new “wonder” drug?

Tight physiological glycaemic homoeostasis should be a human right but it has been somewhat hijacked by our need for food “rewards” and the vested interests of the processed food industrial complex. The sooner we return the power to individuals, in the form of continuous glucose monitoring and education on sensible dietary advice for a healthy life, the sooner we may stem the tsunami of morbidity and mortality associated with preventable cardiovascular disease.

## References

[1] Cowie MR. The heart failure epidemic: a UK perspective. Echo Res Pract. 2017;4:R15–20. 10.1530/ERP-16-0043 PMC5435875 28196811

[2] Ponikowski P, Anker SD, AlHabib KF, Cowie MR, Force TL, Hu S (2014). Heart failure: preventing disease and death worldwide. ESC Heart Fail.

[3] McMurray JJV, Solomon SD, Inzucchi SE, Kober L, Kosiborod MN, Martinez FA (2019). Dapagliflozin in patients with heart failure and reduced ejection fraction. N Engl J Med.

[4] Packer M, Anker SD, Butler J, Filippatos G, Pocock SJ, EMPEROR-Reduced Trial Investigators (2020). Cardiovascular and renal outcomes with Empagliflozin in heart failure. N. Engl J Med.

[5] Cherney DZ, Odutayo A, Aronson R, Ezekowitz J, Parker JD (2019). Sodium glucose cotransporter-2 inhibition and cardiorenal protection: JACC review topic of the week. J Am Coll Cardiol.

[6] Cosentino F, Grant PJ, Aboyans V, Bailey CJ, Ceriello A, Delgado V (2020). 2019 ESC guidelines on diabetes, pre-diabetes, and cardiovascular diseases developed in collaboration with the EASD. Eur Heart J.

[7] Unwin DJ, Tobin SD, Murray SW, Delon C, Brady AJ (2019). Substantial and sustained improvements in blood pressure, weight and lipid profiles from a carbohydrate restricted diet: an observational study of insulin resistant patients in primary care. Int J Environ Res Public Health.

[8] Zinman B, Wanner C, Hantel S, Mattheus M, Devins T, Broedl UC (2015). Empagliflozin, cardiovascular outcomes, and mortality in type 2 diabetes. N. Engl J Med.

[9] Neal B, Perkovic V, Matthews DR (2017). Canagliflozin and cardiovascular and renal events in type 2. Diabetes N Engl J Med.

[10] Fioretto P, Zambon A, Rossato M, Busetto L, Vettor R (2016). SGLT2 inhibitors and the diabetic kidney. Diabetes Care.

[11] De Albuquerque Rocha N, Neeland IJ, McCullough PA, Toto RD, McGuire DK (2018). Effects of sodium glucose co-transporter 2 inhibitors on the kidney. Diab Vasc Dis Res..

[12] Nakamura N, Matsui T, Ishibashi Y, Yamagishi SI (2015). Insulin stimulates SGLT2-mediated tubular glucose absorption via oxidative stress generation. Diabetol Metab Syndr..

[13] Bray GA (2003). Low-carbohydrate diets and realities of weight loss. JAMA.

[14] Palmer LG, Schnermann J (2015). Integrated control of Na transport along the nephron. CJASN..

[15] Brady JA, Hallow KM (2018). Model-based evaluation of proximal sodium reabsorption through SGLT2 in health and diabetes and the effect of inhibition with canagliflozin. J Clin Pharmacol.

[16] Song P, Onishi A, Koepsell H, Vallon V (2016). Sodium glucose cotransporter SGLT1 as a therapeutic target in diabetes mellitus. Expert Opin Therapeutic Targets..

[17] DeFronzo RA, Norton L (2017). Abdul-Ghani M. Renal, metabolic and cardiovascular considerations of SGLT2 inhibition. Nat Rev Nephrol.

[18] Pessoa TD, Campos LCG, Carraro-Lacroix L, Girardi ACC, Malnic G (2014). Functional role of glucose metabolism, osmotic stress, and sodium-glucose cotransporter isoform-mediated transport on Na+/H + exchanger isoform 3 activity in the renal proximal tubule. JASN..

[19] Ferrannini E, Baldi S, Frascerra S, Astiarraga B, Barsotti E, Clerico A (2017). Renal handling of ketones in response to sodium-glucose cotransporter 2 inhibition in patients with type 2 diabetes. Diabetes Care.

[20] Ferrannini E, Baldi S, Frascerra S, Astiarraga B, Heise T, Bizzotto R (2016). Shift to fatty substrate utilization in response to sodium-glucose cotransporter 2 inhibition in subjects without diabetes and patients with type 2 diabetes. Diabetes.

[21] Abdul-Ghani M, Del Prato S, Chilton R, DeFronzo RA (2016). SGLT2 inhibitors and cardiovascular risk: lessons learned from the EMPA-REG OUTCOME study. Diabetes Care.

[22] Ferrannini G, Hach T, Crowe S, Sanghvi A, Hall KD, Ferrannini E (2015). Energy balance after sodium-glucose cotransporter 2 inhibition. Diabetes Care.

[23] Cabo Rafaelde, Mattson MarkP (2019). Effects of intermittent fasting on health, aging, and disease December 26, 2019. N. Engl J Med.

[24] Sävendahl L, Underwood LE (1999). Fasting increases serum total cholesterol, LDL cholesterol and apolipoprotein B in healthy, nonobese humans. J Nutr..

[25] Mattson MP, Longo VD, Harvie M (2017). Impact of intermittent fasting on health and disease processes. Ageing Res Rev..

[26] Parthasarathy S, Raghavamenon A, Garelnabi MO, Santanam N (2010). Oxidized low-density lipoprotein. Methods Mol Biol.

[27] Toth PP (2019). Lipoprotein subfractions in patients with acute coronary syndromes: should we reach beyond LDL-C?. Curr Vasc Pharmacol.

[28] Ness GC, Zhao Z, Wiggins L (1994). Insulin and glucagon modulate hepatic 3-hydroxy-3-methylglutaryl-coenzyme A reductase activity by affecting immunoreactive protein levels. J Biol Chem..

[29] Park YM, Kashyap RS, Major AJ, Silverstein RL (2012). Insulin promotes macrophage foam cell formation: potential implications in diabetes-related atherosclerosis. Lab Invest..

[30] Mudaliar S, Alloju S, Henry RR (2016). Can a shift in fuel energetics explain the beneficial cardiorenal outcomes in the EMPA-REG OUTCOME study? A unifying hypothesis. Diabetes Care.

[31] Dei Cas A, Khan SS, Butler J (2015). Impact of diabetes on epidemiology, treatment, and outcomes of patients with heart failure. JACC Heart Fail.

[32] Wilcox G (2005). Insulin and insulin resistance. Clin Biochem Rev.

[33] Kelley DE, Mandarino LJ (2000). Fuel selection in human skeletal muscle in insulin resistance: a reexamination. Diabetes.

[34] Mather KJ, Hutchins GD, Perry K (2016). Assessment of myocardial metabolic flexibility and work efficiency in human type 2 diabetes using 16-[18F]fluoro-4-thiapalmitate, a novel PET fatty acid tracer. Am J Physiol Endocrinol Metab.

[35] Cherian S, Lopaschuk GD, Carvalho E (2012). Cellular cross-talk between epicardial adipose tissue and myocardium in relation to the pathogenesis of cardiovascular disease. Am J Physiol Endocrinol Metab.

[36] Ritchie RH (2009). Evidence for a causal role of oxidative stress in the myocardial complications of insulin resistance. Heart Lung Circ.

[37] Mccormack JG, Barr RL, Wolff AA, Lopaschuk GD (1996). Ranolazine stimulates glucose oxidation in normoxic, ischemic, and reperfused ischemic rat hearts. Circulation.

[38] Karwi QG, Uddin GM, Ho KL, Lopaschuk GD (2018). Loss of metabolic flexibility in the failing heart. Front Cardiovasc Med..

[39] Saslow L, Summer C, Aitken J, Unwin D (2018). Outcomes of a digitally delivered low-carbohydrate type 2 diabetes self-management program: 1-year results of a single-arm longitudinal study. JMIR Diabetes.

[40] Bhanpuri N, Hallberg S, Williams P (2018). Cardiovascular disease risk factor responses to a type 2 diabetes care model including nutritional ketosis induced by sustained carbohydrate restriction at 1 year: an open label, non-randomized, controlled study. Cardiovasc Diabetol.

[41] Lean ME, Leslie WS, Barnes A (2018). Two-year results of the randomised Diabetes Remission Clinical Trial (DiRECT). Lancet.

